# Supplementary material for: Iterative improvement in the automatic modular design of robot swarms
Source: PeerJ Comput Sci. 2020 Dec 7;6:e322. doi: 10.7717/peerj-cs.322 (PMC7924708; doi:10.7717/peerj-cs.322)
Supplement: Supplemental Information 3 [file peerj-cs-06-322-s003.zip › argos3/doc/api/standalone/a00627_source.html]

ARGoS: plugins/simulator/physics\_engines/pointmass3d/pointmass3d\_quadrotor\_model.cpp Source File


- Main Page
- Related Pages
- Namespaces
- Classes
- Files

- File List
- File Members

# plugins/simulator/physics\_engines/pointmass3d/pointmass3d\_quadrotor\_model.cpp

Go to the documentation of this file.

```
00001 
00007 #include "pointmass3d_quadrotor_model.h"
00008 #include <argos3/core/utility/logging/argos_log.h>
00009 #include <argos3/core/utility/math/cylinder.h>
00010 #include <argos3/core/simulator/simulator.h>
00011 #include <argos3/core/simulator/space/space.h>
00012 #include <argos3/plugins/simulator/physics_engines/pointmass3d/pointmass3d_engine.h>
00013 
00014 namespace argos {
00015 
00016    static Real SymmetricClamp(Real f_max,
00017                               Real f_value) {
00018       if(f_value >  f_max) return  f_max;
00019       if(f_value < -f_max) return -f_max;
00020       return f_value;
00021    }
00022 
00023    /****************************************/
00024    /****************************************/
00025 
00026    CPointMass3DQuadRotorModel::CPointMass3DQuadRotorModel(CPointMass3DEngine& c_engine,
00027                                                           CEmbodiedEntity& c_body,
00028                                                           CQuadRotorEntity& c_quadrotor,
00029                                                           Real f_body_height,
00030                                                           Real f_arm_length,
00031                                                           Real f_body_mass,
00032                                                           Real f_body_inertia,
00033                                                           const CVector3& c_pos_kp,
00034                                                           const CVector3& c_pos_kd,
00035                                                           Real f_yaw_kp,
00036                                                           Real f_yaw_kd,
00037                                                           const CVector3& c_vel_kp,
00038                                                           const CVector3& c_vel_kd,
00039                                                           Real f_rot_kp,
00040                                                           Real f_rot_kd,
00041                                                           const CVector3& c_max_force,
00042                                                           Real f_max_torque) :
00043       CPointMass3DModel(c_engine, c_body),
00044       m_cQuadRotorEntity(c_quadrotor),
00045       m_fBodyHeight(f_body_height),
00046       m_fArmLength(f_arm_length),
00047       m_fBodyMass(f_body_mass),
00048       m_fBodyInertia(f_body_inertia),
00049       m_cPosKP(c_pos_kp),
00050       m_cPosKD(c_pos_kd),
00051       m_fYawKP(f_yaw_kp),
00052       m_fYawKD(f_yaw_kd),
00053       m_cVelKP(c_vel_kp),
00054       m_cVelKD(c_vel_kd),
00055       m_fRotKP(f_rot_kp),
00056       m_fRotKD(f_rot_kd),
00057       m_cMaxForce(c_max_force),
00058       m_fMaxTorque(f_max_torque) {
00059       Reset();
00060       /* Register the origin anchor update method */
00061       RegisterAnchorMethod(GetEmbodiedEntity().GetOriginAnchor(),
00062                            &CPointMass3DModel::UpdateOriginAnchor);
00063       /* Get initial rotation */
00064       CRadians cTmp1, cTmp2;
00065       GetEmbodiedEntity().GetOriginAnchor().Orientation.ToEulerAngles(m_cYaw, cTmp1, cTmp2);
00066    }
00067 
00068    /****************************************/
00069    /****************************************/
00070 
00071    void CPointMass3DQuadRotorModel::Reset() {
00072       CPointMass3DModel::Reset();
00073       m_pfLinearError[0] = 0.0f;
00074       m_pfLinearError[1] = 0.0f;
00075       m_pfLinearError[2] = 0.0f;
00076       m_fRotError = 0.0f;
00077    }
00078 
00079    /****************************************/
00080    /****************************************/
00081 
00082    void CPointMass3DQuadRotorModel::UpdateFromEntityStatus() {
00083       m_sDesiredPositionData = m_cQuadRotorEntity.GetPositionControlData();
00084    }
00085 
00086    /****************************************/
00087    /****************************************/
00088 
00089    void CPointMass3DQuadRotorModel::Step() {
00090       /*
00091        * Update positional information
00092        */
00093       /* Integration step */
00094       m_cPosition += m_cVelocity * m_cPM3DEngine.GetPhysicsClockTick();
00095       m_cYaw      += m_cRotSpeed * m_cPM3DEngine.GetPhysicsClockTick();
00096       /* Limit the quad-rotor position within the arena limits */
00097       const CRange<CVector3>& cLimits = CSimulator::GetInstance().GetSpace().GetArenaLimits();
00098       m_cPosition.SetX(
00099          Min(Max(m_cPosition.GetX(),
00100                  cLimits.GetMin().GetX() + m_fArmLength),
00101              cLimits.GetMax().GetX() - m_fArmLength));
00102       m_cPosition.SetY(
00103          Min(Max(m_cPosition.GetY(),
00104                  cLimits.GetMin().GetY() + m_fArmLength),
00105              cLimits.GetMax().GetY() - m_fArmLength));
00106       m_cPosition.SetZ(
00107          Min(Max(m_cPosition.GetZ(),
00108                  cLimits.GetMin().GetZ()),
00109              cLimits.GetMax().GetZ() - m_fBodyHeight));
00110       /* Normalize the yaw */
00111       m_cYaw.UnsignedNormalize();
00112       /*
00113        * Update velocity information
00114        */
00115       m_cVelocity += (m_cPM3DEngine.GetPhysicsClockTick() / m_fBodyMass)    * m_cAcceleration;
00116       m_cRotSpeed += (m_cPM3DEngine.GetPhysicsClockTick() / m_fBodyInertia) * m_cTorque;
00117       /*
00118        * Update control information
00119        */
00120       if(m_cQuadRotorEntity.GetControlMethod() == CQuadRotorEntity::POSITION_CONTROL)
00121          PositionalControl();
00122       else
00123          SpeedControl();
00124       /*
00125        * Update force/torque information
00126        */
00127       m_cAcceleration.SetX(m_cLinearControl.GetX());
00128       m_cAcceleration.SetY(m_cLinearControl.GetY());
00129       m_cAcceleration.SetZ(m_cLinearControl.GetZ() + m_fBodyMass * m_cPM3DEngine.GetGravity());
00130       m_cTorque.SetValue(m_fRotationalControl);
00131    }
00132 
00133    /****************************************/
00134    /****************************************/
00135 
00136    void CPointMass3DQuadRotorModel::CalculateBoundingBox() {
00137       GetBoundingBox().MinCorner.Set(
00138          GetEmbodiedEntity().GetOriginAnchor().Position.GetX() - m_fArmLength,
00139          GetEmbodiedEntity().GetOriginAnchor().Position.GetY() - m_fArmLength,
00140          GetEmbodiedEntity().GetOriginAnchor().Position.GetZ());
00141       GetBoundingBox().MaxCorner.Set(
00142          GetEmbodiedEntity().GetOriginAnchor().Position.GetX() + m_fArmLength,
00143          GetEmbodiedEntity().GetOriginAnchor().Position.GetY() + m_fArmLength,
00144          GetEmbodiedEntity().GetOriginAnchor().Position.GetZ() + m_fBodyHeight);
00145    }
00146 
00147    /****************************************/
00148    /****************************************/
00149 
00150    bool CPointMass3DQuadRotorModel::CheckIntersectionWithRay(Real& f_t_on_ray,
00151                                                              const CRay3& c_ray) const {
00152       CCylinder m_cShape(m_fArmLength,
00153                          m_fBodyHeight,
00154                          m_cPosition,
00155                          CVector3::Z);
00156       return m_cShape.Intersects(f_t_on_ray, c_ray);
00157    }
00158    
00159    /****************************************/
00160    /****************************************/
00161 
00162    void CPointMass3DQuadRotorModel::PositionalControl() {
00163       /* Linear control */
00164       m_cLinearControl.Set(
00165          SymmetricClamp(m_cMaxForce.GetX(), PDControl(
00166                            m_sDesiredPositionData.Position.GetX() - m_cPosition.GetX(),
00167                            m_cPosKP.GetX(),
00168                            m_cPosKD.GetX(),
00169                            m_pfLinearError[0])),
00170          SymmetricClamp(m_cMaxForce.GetY(), PDControl(
00171                            m_sDesiredPositionData.Position.GetY() - m_cPosition.GetY(),
00172                            m_cPosKP.GetY(),
00173                            m_cPosKD.GetY(),
00174                            m_pfLinearError[1])),
00175          SymmetricClamp(m_cMaxForce.GetZ(), PDControl(
00176                            m_sDesiredPositionData.Position.GetZ() - m_cPosition.GetZ(),
00177                            m_cPosKP.GetZ(),
00178                            m_cPosKD.GetZ(),
00179                            m_pfLinearError[2]) - m_fBodyMass * m_cPM3DEngine.GetGravity()));
00180       /* Rotational control */
00181       m_fRotationalControl =
00182          SymmetricClamp(m_fMaxTorque, PDControl(
00183                            (m_sDesiredPositionData.Yaw - m_cYaw).SignedNormalize().GetValue(),
00184                            m_fYawKP,
00185                            m_fYawKD,
00186                            m_fRotError));
00187    }
00188 
00189    /****************************************/
00190    /****************************************/
00191 
00192    void CPointMass3DQuadRotorModel::SpeedControl() {
00193       /* Linear control */
00194       m_cLinearControl.Set(
00195          SymmetricClamp(m_cMaxForce.GetX(), PDControl(
00196                            m_sDesiredSpeedData.Velocity.GetX() - m_cVelocity.GetX(),
00197                            m_cVelKP.GetX(),
00198                            m_cVelKD.GetX(),
00199                            m_pfLinearError[0])),
00200          SymmetricClamp(m_cMaxForce.GetY(), PDControl(
00201                            m_sDesiredSpeedData.Velocity.GetY() - m_cVelocity.GetY(),
00202                            m_cVelKP.GetY(),
00203                            m_cVelKD.GetY(),
00204                            m_pfLinearError[1])),
00205          SymmetricClamp(m_cMaxForce.GetZ(), PDControl(
00206                            m_sDesiredSpeedData.Velocity.GetZ() - m_cVelocity.GetZ(),
00207                            m_cVelKP.GetZ(),
00208                            m_cVelKD.GetZ(),
00209                            m_pfLinearError[2]) - m_fBodyMass * m_cPM3DEngine.GetGravity()));
00210       /* Rotational control */
00211       m_fRotationalControl =
00212          SymmetricClamp(m_fMaxTorque, PDControl(
00213                            (m_sDesiredSpeedData.RotSpeed - m_cRotSpeed).GetValue(),
00214                            m_fRotKP,
00215                            m_fRotKD,
00216                            m_fRotError));
00217    }
00218 
00219    /****************************************/
00220    /****************************************/
00221 
00222    Real CPointMass3DQuadRotorModel::PDControl(Real f_cur_error,
00223                                               Real f_k_p,
00224                                               Real f_k_d,
00225                                               Real& f_old_error) {
00226       Real fOutput =
00227          f_k_p * f_cur_error +                                                      /* proportional term */
00228          f_k_d * (f_cur_error - f_old_error) / m_cPM3DEngine.GetPhysicsClockTick(); /* derivative term */
00229       f_old_error = f_cur_error;
00230       return fOutput;
00231    }
00232 
00233    /****************************************/
00234    /****************************************/
00235 
00236    void CPointMass3DQuadRotorModel::UpdateOriginAnchor(SAnchor& s_anchor) {
00237       s_anchor.Position = m_cPosition;
00238       s_anchor.Orientation = CQuaternion(m_cYaw, CVector3::Z);
00239    }
00240 
00241    /****************************************/
00242    /****************************************/
00243 
00244 }
```

---

Generated on 10 Jul 2018 for ARGoS by 
 1.6.1
